# Value incoherence precedes value change: Evidence from value development in childhood and adolescence across cultures

**DOI:** 10.1177/08902070241289969

**Published:** 2024-11-19

**Authors:** Ella Daniel, Anat Bardi, Julie A Lee, Ricarda Scholz-Kuhn, Einat Elizarov, Jan Cieciuch, Ariel Knafo-Noam, Alice Ramos, Michele Vecchione, Rene Algesheimer, Evelia Murcia Alvarez, Avital Ben Dror Lankry, Maya Benish-Weisman, Ricardo Borges Rodrigues, Anat Chomsky, Patricia R. Collins, Eldad Davidov, Anna K Döring, Stefanie Habermann, Dana Katsoty, Martin Kindschi, Elena Makarova, Gilda Marsicano, Kinneret Misgav, Thomas P Oeschger, Leonor Pereira da Costa, Joanne Sneddon, Iva Tendais, Louise Twito -Weingarten

**Affiliations:** 1 26745 Tel Aviv University, Tel Aviv, Israel; 2 3162 Royal Holloway University of London, Egham, UK; 3 2720 University of Western Australia, Perth, WA, Australia; 4 27209 University of Basel, Basel, Switzerland; 5 26748 University of Haifa, Haifa, Israel; 6 27217 Cardinal Stefan Wyszynski University in Warsaw, Warsaw, Poland; 7 University of Zurich, Zurich, Switzerland; 8 26742 The Hebrew University of Jerusalem, Jerusalem, Israel; 9 37809 Instituto de Ciências Sociais da Universidade de Lisboa, Lisboa, Portugal; 10 9311 Sapienza University of Rome, Rome, Italy; 11 27217 University of Zurich, Zurich, Switzerland; 12 37809 Instituto Universitário de Lisboa (ISCTE-IUl), Lisboa, Portugal; 13 2498 Edith Cowan University, Joondalup, WA, Australia; 14 27217 University of Cologne, Cologne, Germany; 15 4921 University of Westminster, London, UK; 16 70887 Universidade Lusófona de Humanidades e Tecnologias (ULHT), Lisboa, Portugal

**Keywords:** personality coherence, value change, value structure, childhood, adolescence

## Abstract

We test the theory that personality incoherence may instigate personality change in the context of personal values. Values’ near-universal organization makes value incoherence assessment straightforward. The study included 13 longitudinal samples from seven cultures (Australia, Israel Palestinian citizens, Israel Jewish majority, Italy, Poland, Portugal, and Switzerland), total *N* = 7,126, and T1 *M*_*age*_ ranging between 6 and 18. Each participant reported values between two- and six-times. Using unfolding analysis, we calculated the fit of the internal value structure of each participant at the first time point to the value structure in their sample (normative structure) and to the theoretical structure of values. We estimated value change using Growth Curve Modeling (when at least three measurement times were available) and the difference between T1 and T2 in each sample. We correlated value incoherence with value change and estimated the effect across samples using a meta-analysis. Incoherence with the structure of values predicted greater value change. The associations were stronger when participant’s value structures were compared to the normative value structure at T1 than when they were compared to the theoretical structure. A meta-regression analysis indicated that effects were not moderated by age. We discuss possible underlying processes and implications for personality development.

## Plain language summary

Researchers have suggested that internal conflicts are difficult to endure, and individuals whose personality is chaotic will be less able to function. As a result, they will be more likely to change their personality. We test this prediction for one aspect of a person’s personality: their values. Values are personal goals, defining what an individual finds most important in life. We assembled 13 samples from seven different cultures, including 7,126 children and adolescents (6–18 years of age). We calculated the internal coherence of their values at time 1, to identify disorganization or internal conflicts. We then followed the participants over time, to discover whether their value priorities changed. Our analysis shows that children and adolescents who reported disorganized values at the beginning of the study changed their value priorities over time. Thus, individuals who find it hard to decide what they find important in life, holding different competing goals, are more likely to shift between goals.

## Introduction

Personality development is characterized, among other processes, by an increase in self-coherence (Caspi & Shiner, 2006). Across theories, psychologists suggest that individuals aspire for coherence in personality, that is, internal integration and unity (Fajkowska, 2023; Fournier et al., 2015). Moreover, incoherence, or conflict between personality aspects, may lead to difficulty to function, that is, to achieve self-growth and action control. For that reason, such incoherence should, theoretically, drive personality change (Kuhl et al., 2015; Quirin & Kuhl, 2022) and coherence will be a marker of maturation (Fournier et al., 2022). We offer a direct test of the theory by focusing on inter-relations within a full system of one personality aspect, that is, personal values. We provide the first evidence for the idea that personality incoherence drives personality development. The evidence we provide is thorough, in that it includes thirteen longitudinal samples of value development in children and adolescents from multiple cultures.

The personality construct includes one’s traits, personal narratives, and guiding motivations. Within this construct, values are a key aspect, defining the typical motivations driving individuals in their lives (McAdams, 2013; Roberts & Yoon, 2022; Rokeach, 1973; Schwartz, 1992). The well-validated Schwartz Personal Values Theory (Borg et al., 2017; Cieciuch et al., 2014; Fontaine et al., 2008; Schwartz, 1992) offers a clear benchmark to a coherent value system, based on value inter-relations. This system includes inherent conflicts and compatibilities, such that conflicting values are typically difficult to pursue simultaneously, while compatible values that share similar motivations may be satisfied by similar pursuits. This system of compatible and conflicting values provides a clear illustration of coherence versus incoherence in personality. Hence, focusing on personal values enables us to empirically test whether change is more likely under conditions of incoherence in the organization of personality.

To test whether incoherence in the structure of values precedes value change, we need to investigate a population undergoing value change. Previous research has shown that values of adults are highly stable and rarely change (Leijen et al., 2022; Schuster et al., 2019). In contrast, the values of children and adolescents change substantially as they grow (Cieciuch et al., 2016; Daniel & Benish-Weisman, 2019; Tamm & Tulviste, 2022; Vecchione et al., 2020). We focus our examination on children and adolescents from multiple cultures, to investigate possible cross-cultural similarities and differences in the process. Further, the use of a wide range of age-groups (from middle childhood to late adolescence) enables us to test whether this phenomenon occurs across youth or peaks during adolescence (Erikson, 1968).

### Personal values

Personal values (e.g., caring for others, success, humility, and curiosity) are abstract motivational goals that individuals see as worth pursuing and want to achieve in life (e.g., Schwartz, 1992). These basic motivational goals are used as standards for the evaluation of attitudes and behaviors. They motivate social behaviors (Sagiv & Roccas, 2021), such as prosociality (Abramson et al., 2018; Benish-Weisman et al., 2019; Misgav et al., 2022; Sagiv et al., 2011), aggression (Benish-Weisman, 2019), and health behaviors (Nieh et al., 2018; Piko, 2005). Values are at the core of one’s identity, providing individuals with a sense of self-knowledge and clarity (Hitlin & Piliavin, 2004).

Values have been identified among children as early as five years of age (Abramson et al., 2018; Elizarov et al., 2023; Lee et al., 2017), using age-appropriate measures. That is, children can coherently report on the importance of their values in response to concrete questionnaires, depicting children engaged in a variety of value-consistent behaviors (Collins et al., 2017; Döring et al., 2015). The values they report are expressed in mostly concrete and observable terms (Misgav & Daniel, 2022; Misgav et al., 2023; Shachnai & Daniel, 2020). Adolescence, in contrast, has often been considered a hallmark of value formation; a time of change and development in the importance of values (Daniel & Benish-Weisman, 2019). Further, children and adolescents’ values are associated with their behavior, as observed in an experimental setting, or as rated by themselves and by their peers, within and across time (Abramson et al., 2018; Benish-Weisman, 2015; Daniel et al., 2020; Misgav et al., 2022; Vecchione et al., 2016). Thus, the value priorities of children appear to be meaningful in their lives.

### Personality and value coherence

Individuals’ personality is a complex structure, including their traits, goals, and life stories (McAdams, 2013). Given its complexity, it is often found to include fragmentations and conflicts. Personality coherence defines the level of integration and coordination across personality aspects. Those may include coherence in one’s traits, in the recollection of the past, and importantly, in one’s goals (Fournier et al., 2015). Such coherence suggests that an individual holds a unified sense of direction and purpose. That is, one’s goals and striving help bring each other about or even promote the achievement of higher-order goals (Fournier et al., 2015; Sheldon & Kasser, 1995). High personality coherence was associated with well-being, autonomy, and growth (Fournier et al., 2022), as well as improved goal-pursuit (Quirin & Kuhl, 2022).

**Figure 1.**
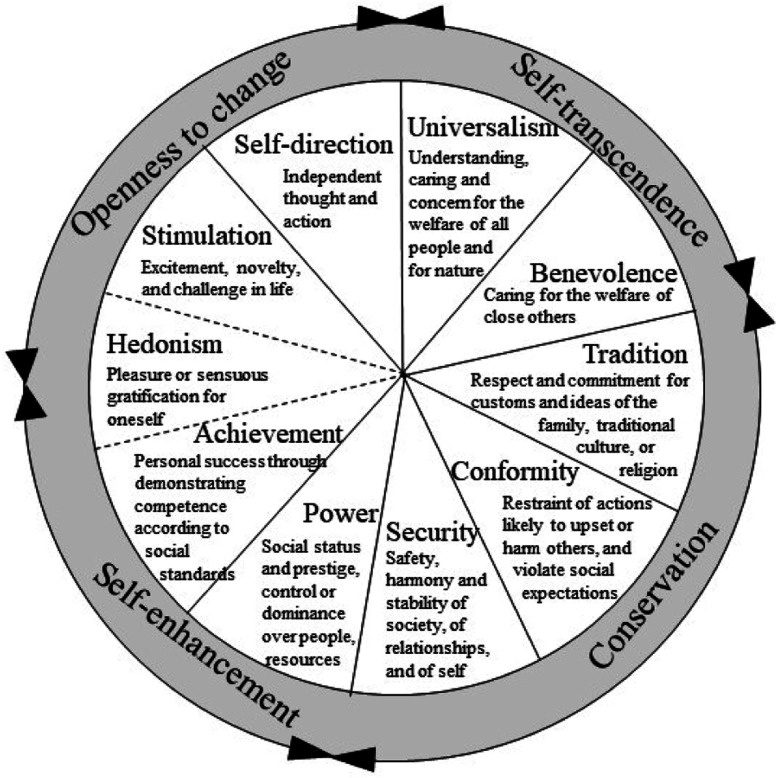
Definitions and structure of values (Schwartz, 1992).

Organization of life goals can easily be conceptualized in the context of the Personal Values Theory (Schwartz, 1992). The theory identifies 10 basic values—self-direction, stimulation, hedonism, achievement, power, security, conformity, tradition, benevolence, and universalism. These values can be organized as four higher-order value dimensions that summarize the associations among them: openness to change, conservation, self-enhancement, and self-transcendence. These associations are a fundamental feature of the values theory, suggesting that values are not merely a list of unrelated motivations but hold complex, systematic associations among them. These associations can be represented as a circular motivational continuum (see Figure 1). Motivations driving and directing each value are inherently compatible with motivations driving and directing neighboring values in the circle but stand in conflict with the motivations driving and directing opposing values in the circle. Hence, the pursuit of one value leads to consequences that match some values but contradict others. For example, self-direction values are directed toward independence, creativity, and curiosity. Children who pursue these values may invent decorations for their room or maintain their opinion even if other children do not agree (Vecchione et al., 2016). These actions are also compatible with the pursuit of neighboring stimulation values, which are directed toward experiencing change and variability. In contrast, conformity values are directed toward preserving the status quo and restricting behaviors and thoughts to those that adhere to norms and expectations. Thus, conformity values conflict with the pursuit of the opposing self-direction values. Research shows that placing similar importance on very different values may be associated with a subjective experience of conflict (Bouckenooghe et al., 2005; Sverdlik, 2012).

Hundreds of studies have confirmed the existence of the circular structure of values in varied samples across cultures, as evidenced by inter-relations among value priorities (Sagiv et al., 2017; Sagiv & Schwartz, 2022; Schwartz, 2012), and in studies of reaction times, memory accessibility, and activation of brain regions to resolve conflicts (Leszkowicz et al., 2017; Maio, 2010; Pakizeh et al., 2007). However, some deviations in the structure were identified. These deviations were sometimes attributed to random variance. In other cases, studies identified systematic differences in the structure that attest to differences between cultures in the meaning of values (Bilsky et al., 2011; Fontaine et al., 2008; Rudnev et al., 2018).

The organization of the value system has typically been investigated *between* individuals, at the sample level. These results indicated that if individuals hold one value as more important than other individuals within the sample, they are also likely to hold the conflicting value as less important than other individuals within the sample (Fontaine et al., 2008; Skimina et al., 2021). However, the theory of personal values suggests that the value structure exists not only at the sample level but also at the individual level. Recent developments have enabled the testing of the structure of values *within* individuals (Borg et al., 2017; Lee et al., 2017; Skimina et al., 2021). Across studies, findings indicate that the within-individual structure of adults’ values resembles the theoretical structure postulated by Schwartz (1992), where each individual is likely to hold compatible values as similarly important and conflicting values as less important (Borg et al., 2017; Skimina et al., 2021).

**Figure 2.**
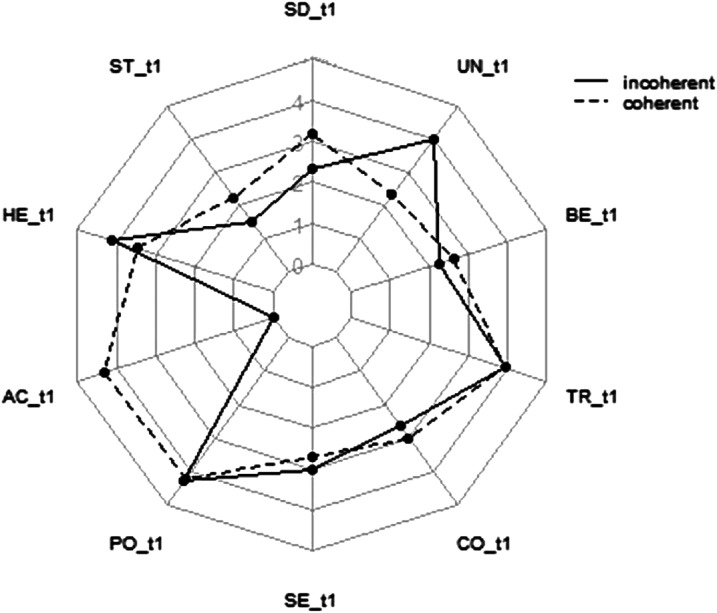
Incoherent versus coherent value priorities of two individuals.

Figure 2 depicts a set of incoherent versus coherent value priorities of two individuals selected from the current sample. It demonstrates that incoherent value systems include adjacent values of different importance and contrasting values of similar importance. It also demonstrates that coherent value systems include adjacent values of similar importance and contrasting values of different importance.

The value structure has also been investigated among children and adolescents. At the sample level, the between-individual value structure, similar to the one found in adulthood, was already identified in children aged five to seven (Abramson et al., 2018; Berson & Oreg, 2016; Bilsky et al., 2013; Döring et al., 2015; Lee et al., 2017; for a review see Knafo-Noam et al., 2024). It was also identified among adolescents (Daniel & Benish-Weisman, 2019; Vecchione et al., 2020). However, there are also some systematic variations by age, with the 10 basic values becoming more differentiated in line with the theoretical structure of values as children approach adolescence (Abramson et al., 2018; Daniel et al., 2020; Lee et al., 2017). Further, studies of individual differences in within-individual value structures found that some children were better described by the theoretical value structure than others (Lee et al., 2017). Importantly, one longitudinal study demonstrated that children became more coherent with the structure from the beginning of middle childhood, as they mature (Daniel et al., 2023).

### Value change

Values are relatively stable characteristics, and value change during adulthood is slow (Daniel et al., 2022; Leijen et al., 2022; Schuster et al., 2019). Individuals tend to maintain values that are adaptive and that support them in functioning within their social conditions and environments. As a result, when adults’ values change, this change is typically slow paced. Some value change was identified as a result of substantial life events, such as immigration or terror attack (Bardi et al., 2014; Cote et al., 2002; Lönnqvist et al., 2013). Intervention, triggering value re-evaluation or changing the accessibility and salience of values, also resulted in value change (for a review, see Russo et al., 2022). However, value change may be short-lived and reversible (Bardi & Goodwin, 2011).

In contrast, during childhood and adolescence, values may change more readily (reviewed in Döring et al., 2016; Knafo-Noam et al., 2024; Twito-Weingarten & Knafo-Noam, 2022), gradually decreasing in the rate of change as they approach adulthood (Daniel & Benish-Weisman, 2019). This is evident in both test–retest associations (Cieciuch et al., 2016; Daniel & Benish-Weisman, 2019; Vecchione et al., 2020) and in change in mean value importance (Cieciuch et al., 2016; Daniel & Benish-Weisman, 2019; Vecchione et al., 2020). These differences in the rate of change may reflect changes in children’s and adolescents’ environment (Benish-Weisman et al., 2022; Daniel, Dys, et al., 2016), their cognitive and socio-cognitive maturation (Misgav & Daniel, 2022; Misgav et al., 2023), or due to having little opportunity in the past to reinforce and entrench their values (Bardi & Goodwin, 2011). Moreover, the task of identity formation, undertaken during adolescence, calls for reconsideration and exploration of value importance (Crocetti, 2017; Meeus, 2018), leading to a higher likelihood of value change. Due to the greater prevalence of value change in childhood and adolescence than adulthood, these periods appear to be promising times to test our proposition regarding value incoherence predicting value change.

### The current investigation

In this paper, we theorize that value incoherence may be associated with further value change. Theory suggests that incoherent personality systems, and specifically goal systems, are a marker of a lack of maturity (Fournier et al., 2022). They hinder functioning, including self-growth and action control (Quirin & Kuhl, 2022). As a result, we can hypothesize that to reach higher levels of maturity and functioning, individuals of low personality coherence are likely to show personality change. As a result, the aim of the current research was to investigate the association between value incoherence and value development in youth. We hypothesize that the less coherent the organization of children’s and adolescent’s values are, the more likely they are to change their value priorities over time. We investigated this proposition both in terms of deviation from (1) the normative structure of values in each specific sample at Time 1 and (2) the theoretical structure of values (Schwartz, 1992). Deviation from the normative structure of values in each sample was examined because prior research has found some differences in the structure of values across cultures (Bilsky et al., 2011; Fontaine et al., 2008; Rudnev et al., 2018) and age groups (Daniel et al., 2023). It is possible that within a population of a particular age in a particular culture, the structure of values reflects specific social norms, and coherence with this specific structure will be especially meaningful in a child’s life. In this case, deviation from the theoretical value structure might not drive the child towards change to the same extent as would deviation from the normative value structure in one’s particular age and culture.

## Method

### Participants

The full study was composed of *K* = 13 samples, with a total of *N*_*all*_ _*full*_ = 7126 children and adolescents that reported their values two times or more. Of these, *K* = 9 samples (*n*_*long-term full*_ *=* 5425 children and adolescents) reported their values three times or more. As detailed below, some analyses required the use of participants reporting all information (i.e., with no missing data). In these analyses, the sample reporting their values two time or more was *n*_*all no missing*_ = 4519 and those reporting their values three times or more was *n*_*long-term no missing*_ = 4034. The samples included middle childhood (T1 *M*_*age*_ was between 6 and 8, *K* = 4, *n*_*all full*_ = 2,432, *n*_*all no missing*_ = 1464), late childhood (T1 *M*_*age*_ was between 8.5 and 11, *K* = 6, *n*_*all full*_ = 2,038, *n*_*all no missing*_ = 1493), and adolescence (T1 *M*_*age*_ was between 13 and 15, *K* = 3, *n*_*all full*_ = 2,656, *n*_*all no missing*_ = 1562; see Table 1 for further details). Samples were collected in seven cultural groups and six countries: Australia, Israel Jewish majority, Israel Palestinian citizens, Italy, Poland, Portugal, and Switzerland. Samples varied in terms of sample size, ranging between *n* = 188 and *n* = 1999. Information regarding age characteristics and percent of females in each sample is presented in Table 1. All investigators who published results based on value importance of children or adolescents using a longitudinal design, to the best of the authors’ knowledge at the time of analysis, were invited to participate.

**Table 1.** Sample information.

Country	Age group	N all full sample	N long-term all	N all no missing	N long-term no missing	Age mean	Age SD	Age range	%Female	Time points	App. time gap	Measure	Years
Australia	Middle childhood	587		365		6.57	1.10	5–8	49.74	2	2 Y	AVI-r	2016–2018
Australia	Late childhood	452		161		10.22	0.98	9–12	51.99	2	2 Y	AVI-r	2016–2018
Israel Palestinian citizens	Adolescence	389	389	268	325	13.70	0.50	12–15	54.50	3	1 Y	PVQ40	2011–2014
Israel Jewish majority	Middle childhood	300	300	144	265	7.25	0.64	5.83–8.83	53.69	3	1 Y	PVQ40	2019–2022*
Israel Jewish majority	Late childhood	352		346		8.81	0.36	8–9.99	57.67	2	2 Y	PBVS-C	2013–2017
Israel Jewish majority	Adolescence	268	268	174	223	13.84	0.55	12–15	47.57	3	1 Y	PVQ40	2011–2014
Italy	Late childhood	382	382	282	310	10.67	0.58	10–13	43.19	6	3–6 M	PVQ40	2012–2014
Poland	Middle childhood	265	265	192	231	6.28	0.57	5–8	50.57	6	3–12 M	PBVS-C (Likert scale)	2015–2018
Poland	Late childhood	354	280	238	280	9.75	0.51	9–11	46.33	6	3–12 M	PBVS-C (Likert scale)	2015–2018
Poland	Adolescence	1999	1999	1120	1382	14.57	1.63	12–18	57.58	6	3–12 M	PVQ-RR 57	2015–2018
Portugal	Late childhood	310		310		10.41	2.28	6–14	53.23	2	7–10 M	PBVS-C	2020–2021*
Switzerland	Middle childhood	1280	1280	763	834	6.83	0.52	5–9	49.37	3	3–4 M	PBVS-C	2021–2022*
Switzerland	Late childhood	188	188	156	184	9.65	0.81	8–12	45.74	3	3–12 M	PBVS-C (Likert scale)	2015–2016

*Note.* Y = years; M = months. AVI-r: Animated Values Instrument - Revised (Lee et al., 2017); PVQ40: Portrait Values Questionnaire (Schwartz et al., 2001); PBVS-C: Picture-Based Value Survey for Children (Döring, 2010); PVQ-RR 57: revised Portrait Values Questionnaire (Schwartz, 2017); *N*_*all*_ _*full sample*_ includes participants studied at least two times; *N*_*long term full sample*_ includes participants studied at least three times; *N*_*all*_ _*no missing sample*_ includes participants studied at least two times who have no missing data at T1; *N*_*long-term no missing*_ _*sample*_ includes participants studied at least three times who have no missing data at T1 or T2. * = Collected during the COVID-19 epidemic.

### Measures

The measures varied between studies, reflecting variation in the age-appropriateness of the measures available to estimate the Schwartz (1992) basic values. Details of type of measure used for each study are reported in Table 1. Sample items from each measure are available in Supplemental Material (SM) 1.

#### Value measurement in the middle and late childhood groups

##### PBVS-C

Children’s value structure and priorities were assessed using the Picture-Based Value Survey for Children (PBVS-C). This instrument was designed to be appropriate to the cognitive developmental level of younger children (available upon request from Döring, 2010) and has been applied across cultures (e.g., Cieciuch et al., 2016; Döring et al., 2015; Uzefovsky et al., 2016). In this measure, the level of abstraction of the values was lowered using pictorial items that visually translate and present values as concrete behaviors in situations (Döring, 2010). Specifically, the PBVS-C comprises 20 caption-accompanied pictures (2 for each of the 10 basic values), in which a gender-neutral main character performs a value-relevant action. Sample items are presented in SM 1. The items are ranked using a forced-choice answer format, between the levels of 5 “*very important*” to 1 “*not at all important*” rated on a 5-point Likert scale. Two items measuring the same value were averaged to compute value scores.

##### AVI-r

Children’s value structure and priorities were also assessed using the revised Animated Values Instrument (AVI-r; available upon request from Lee et al., 2017). This instrument was designed to use with children as young as 5 as it is not dependent on children’s reading ability but rather presents short video clips including verbal, visual, and auditory information that translate and present values in concrete terms.

The AVI-r is a web survey that is based on the best–worst scaling method, which extends paired comparisons to the multiple-choice situation (Louviere et al., 2015). The instrument consists of 21 animations, each describing one value item, organized into 21 subsets, each containing five animations. Each animation is shown five times and compared with each other animation once, based on a balanced incomplete block experimental design. After the children watch the five animations included in each subset, they choose the value animation that is “most like you” and the one that is “least like you.” Children’s value-importance scores are determined using the simple count method (Marley & Louviere, 2005), by subtracting the number of times they chose a value animation as “least like you” from the number of times they chose it as “most like you.” This score is divided by five (i.e., the number of times each animation was shown) to produce an 11-point scale, with scores ranging from −1 to +1, where zero represents the midpoint of the scale and the higher the score, the greater the importance of the value. Items were aggregated to form the 10 value scores.

#### Value measurement in the adolescence and late childhood groups

##### PVQ40 and PVQ-RR 57

Adolescents’ values were assessed using the Portrait Values Questionnaire (PVQ 40, Schwartz et al., 2001) or the Refined Portrait Values Questionnaire (PVQ-RR 57; Schwartz, 2017). It has been demonstrated in previous studies that the PVQ is suitable for use with adolescents (Benish-Weisman et al., 2020; Knafo et al., 2008). Each questionnaire item includes a short verbal portrait describing a person’s life goals or aspirations, with each portrait representing a basic value from Schwartz’s theory (Schwartz et al., 2001). Respondents rate how much they resemble the person described in each item on a 6-point Likert scale (from 1 = “*not at all like me*” to 6 = “*very much like me*”). Respondent’s personal value priorities are estimated through these similarity judgements.

In the PVQ40, each of Schwartz’s (1992) 10 basic values is represented by 3–6 items. In the PVQ-RR 57, each of 19 refined values is represented by 3 items but aggregated to produce the 10 personal values. After controlling for respondents’ response tendencies by centering each of their responses around their average response to all questions on the specific scale (Schwartz, 1992), the relevant items for each of the 10 basic values are aggregated to provide 10 value scores. The higher the score, the greater the importance of the value. A full list of items used in the PVQ40 and PVQ-RR 57 can be found in Schwartz (2021).

### Procedures

All data sets were longitudinal, with data collected at two to six time points, three months to two years apart, between 2011 and 2022. Information regarding the number and spacing of measurement points is provided in Table 1.

Each study was conducted in accordance with the specific requirements of the ethics committees of the universities or the responsible authorities in the different countries. Children were recruited through either schools or families. In school sampling, consent for participation was obtained across levels: from the education system (in some countries) and then from school administration. Only then were consent forms sent to parents, with an option to opt-in or opt-out, depending on the requirements approved by the relevant human ethics committee. In family sampling, parents were approached directly to request opt-in consent. Only upon parental approval, trained researchers approached children, requested their assent for participation, and administered self-report questionnaires, assisting participants when needed. The questionnaires were administered either in group (in schools) or individual settings (in schools and homes).

### Transparency and openness

The design of this study and its analysis was not pre-registered. Data and code to reproduce the analysis are publicly available at the Open Science Framework and can be accessed here: https://doi.org/10.17605/OSF.IO/M82VT. The PVQ40 and PVQ-RR 57 measures for different languages can be found in Schwartz (2021). The PBVS-C and AVI-r can be obtained by request from developers, as detailed above. We report all data exclusions, all manipulations, and all measures in the study.

### Analysis plan

The analysis consisted of four steps (summarized in Table 2). In some of the steps, two options existed to test our propositions, we opted to report both options, and compare the results, as detailed below. As a result, we can gauge the stability of the results versus sensitivity to researcher decisions. The first three steps were conducted within each sample. First, we estimated fit of individuals to the expected value structures. Second, we estimated value change over time. Third, we associated fit and value change. As a last step, we summarized the associations across samples.

**Table 2.** Summary of analysis plan.

Analysis	Goal	Output
1a. Normative structure unfolding	Estimate fit of individuals to the value structure within their sample	Alienation coefficient *K*
1b. Theoretical unfolding	Estimate fit of individuals to the theoretical value structure	Alienation coefficient *K*
2a. Latent growth curve modeling	Estimate value change across multiple points in time (3 or more)	Variation in value change (slope)
		Absolute maximal value change (slope)
2b. Difference between values at T2 versus T1	Estimate value change across two points in time	Variation in value change (delta)
		Absolute maximal value change (delta)
3. Pearson correlations	Investigate whether fit of individuals with the structure (steps 1 and 2) is associated with later value change (steps 3 and 4)	Pearson correlations per sample, for each measure of fit and change
4. Random-effects meta-analysis	Estimate the associations of fit to the structure and value change across samples	Pooled effects and variability, for each measure of fit and change

#### Estimation of fit of individuals to the value structures

In the first step, we estimated the within-individual structure of values using unfolding analysis (Borg et al., 2017, 2018), a technique based on Coombs (1964) theory of preferential choice, only recently implemented in the context of values. We used the “smacof” package in R to estimate the models (de Leeuw & Mair, 2009). In the context of values, unfolding analysis translates the preferences of individuals among values, into a 2-dimensional unfolding plot, composed of two layers: the values and the individuals. The model claims that the value preferences of each individual can be represented as a psychological map within the 2-dimensional plot. Given that the Schwartz (1992) theory posits that neighboring values in the circle share similar motivations and opposing values have conflicting motivations, we might expect the unfolding plot to take the form of a circle of values, with the individual located within its bounds. However, this will only be the case, if the circle represents the value preferences of individuals; that is, only if individuals prioritize values according to the theoretically hypothesized conflicts and compatibilities (Borg et al., 2017).

Further, the exact location of the person-points on the map will be directed by their value profiles. For example, a person who highly values self-direction will be located close to the value-point of self-direction and far from the opposing value point of conformity (Borg et al., 2017). Representing so much in a two-dimensional space necessarily creates a solution that does not describe the values of individuals perfectly. We use an indicator of the model fit to the data that compares the reported value priorities of individuals, to those reflected by the estimated model. This goodness of fit measure is called Stress I and is an estimate of the degree to which the distances in the map differ from the distances between data points (Borg et al., 2018). We compare the normalized stress value of the model to the normalized stress norm, created on the basis of 500 permutations of the data. In the permutations, the observed dissimilarities were randomly permuted within each row of the data matrix (Mair et al., 2016). A Stress I value lower than the 5% permutations quantile suggests that the model fits the data well.

Unfolding analysis can be conducted in two ways (Borg et al., 2018). We conducted unfolding analysis both ways and report them below. The first unfolding analysis (normative model) *estimates the location of the values, based on the value preferences of all individuals in the sample, in a bottom-up process.* Simultaneously, we also estimate the location of each individual relative to the values (location within the circle). The location of the values in the normative model is guided by starting values set to the initial configuration of the theoretical value system but not constrained to it. To assess the solution, we investigate whether the value-points form a circle, and whether the order of the value-points in the circle corresponds with theory. We also assess whether person points are dispersed inside the value circle. Last, we use the stress value for goodness of fit.

The second kind of unfolding analysis (theoretical model) is not only guided by the theoretical value system but also restricted to it (Borg et al., 2018). That is, *the location of values is pre-determined by the theoretical structure according to the Schwartz personal values theory*. The unfolding solution is then describing each individual by their position within this superimposed value circle. Here, goodness of fit is estimated by the location of individuals (expected to be dispersed inside the circle), as well as comparison of stress value to the normalized stress norm, as described above.

In addition to an overall model fit, it is also possible to estimate how well each and every participant’s value profile is represented in the two-dimensional space. This is calculated on the basis of the deviation between their reported value priorities and those reflected by the estimated model. The resulting index is termed alienation coefficient *K* (Daniel et al., 2023). Coefficient *K* estimates the extent of divergence of an individual’s value structure from the value structure in each model. For example, if power values and achievement values are closely located in the unfolding solution, but an individual values achievement to a high extent and power to a low extent, the position of their point in the unfolding space will be in line with their preference for one, but not the other value. The solution will not describe their value preferences accurately, leading to high coefficient *K*. In the normative model, this is divergence from the value structure emerging within a specific sample. In the theoretical model, this is divergence from the theoretical value structure. We use this individual difference indicator as a meaningful variable that reflects how “disorganized” the individual’s internal value structure is, and we hypothesize that such individuals will tend to change more in values than those whose value profile is more coherent.

#### Estimation of value change over time

In the second step, within-individual change in values over time was estimated using two techniques, given differences in the number of time points in each sample. First, for those samples in which values were assessed three or more times, we estimated change in each of the 10 basic values using latent growth curve modeling (Duncan & Duncan, 2009) in the R package “lavaan” 0.6-11 (Rosseel, 2012). Consistent with the modeling literature, models resulting in a comparative fit index (Hu & Bentler, 1999) *CFI* >.90, root mean square error of approximation (Kline, 2011) *RMSEA* <.08, and standardized root mean square residuals (Hu & Bentler, 1999) *SRMR* < .09 were deemed an adequate fit and those resulting in a *CFI* >.95, *RMSEA* <.06, and *SRMR* < .06 were deemed an excellent fit (Schermelleh-Engel et al., 2003). In this technique, the latent linear slope of value change across all time points is the index of change in each value for every individual (*s*). Second, for all samples, we also estimated within-individual change in each of the 10 values as the difference between value importance at T2 and value importance at T1 (*d*).

Two theoretical options for indices of change exist. Again, we calculated both to estimate consistency of the results. For both value slope (*s*) and value difference (*d*), we calculated the absolute maximal change across all ten values. This index is based on the assumption that when the value system is changing, this will be expressed by change in at least one value. Second, we calculated the variance of change across all ten values. This index is based on the assumption that when the value system is changing, the 10 values may change in multiple directions. Mean of change across values was not calculated, as it was theoretically expected to be close to zero for most participants because of different directions of change across conflicting values (Bardi et al., 2014; Daniel & Benish-Weisman, 2019).

#### Association of fit to the structure and value change

We tested the associations between misfit with the value structure (coefficient *K* in the normative and theoretical structure) and value change (absolute maximal and variance of *s* and *d*) at the individual level within each sample using Pearson correlations. The number of correlation coefficients calculated was thus 88: normative/theoretical structure (2) *s/*d* (2) * absolute maximal/variance (2) *sample number (*K* = 9/13).

#### Estimation of associations across samples

We conducted a random-effects meta-analysis using the R package “metaphor” 3.0-2 (Viechtbauer, 2010) to estimate the associations of fit to the structure and value change across samples. We first computed weighted mean effect sizes. We estimated the variability in the effects using Cochran’s *Q*, weighting the differences between individual study effects against the pooled effect across studies; *I*^*2*^, estimating the percentage of variation across studies that is due to heterogeneity rather than chance; and *Tau*^*2*^, estimating the standard deviation of underlying effects across studies. We then used mixed-effects meta-regression models to estimate the moderating role of age group, by comparing the reference group of middle childhood to late childhood and to adolescence.

### Missing data

As longitudinal studies are characterized by attrition, some analyses did not include the full sample. Estimation of fit of individuals to the value structure (coefficient *K*) was only conducted for individuals present at the first time point. Estimation of value change over time (slope) was conducted for samples measured three times or more for all individuals in the sample, using the Maximum Likelihood algorithm to account for missing values. The association between the two measures (coefficient *K* and slope) included only complete pairs. Thus, it was calculated with the *n*_*long-term no missing*_ = 4,034, which included 74% of the relevant participants. Estimation of value change (delta) for the sample estimated at least two times was conducted only when both T1 and T2 values were reported. The association between the two measures again included only complete pairs. It was calculated with the *n*_*full no missing*_ = 4,519, which are 63% of the relevant participants (see Table 1 for *n* per sample across analyses).

## Results

### Preliminary analysis

Results of unfolding analyses were used to estimate misfit to the value structure in the normative (sample-driven) and theoretical (theory-driven) models. We estimated how appropriate the estimated model is to describe the data by comparing the resulting *Stress I* value to a *Stress I* value based on randomly permuted data (Mair et al., 2016). In all samples, the Stress I index was significantly lower than the permutated stress norm (i.e., the mean-permutated stress and the stress level at the lowest 5% of the permutated stress distribution). In only 1 of the 13 samples (Polish middle childhood sample), the theoretical model showed Stress I similar to the lower 5% permutation, indicating that their values were better described by the theory-driven structure than the sample-driven structure. Importantly, the two structures were mostly, although not fully, similar (see Table 3). In all other cases, children report values that adhere to both the normative and theoretical structure of personal values.

**Table 3.** Unfolding analysis stress indicating model fit.

Country	Age group	Normative model			Theoretical model		
		*Stress I*	Mean permutations	5% Permutations quantile	*Stress I*	Mean permutations	5% Permutations quantile
Australia	Middle childhood	0.18	0.22	0.21	0.20	0.22	0.22
Australia	Late childhood	0.19	0.25	0.25	0.24	0.26	0.26
Israel Palestinian citizens	Adolescence	0.20	0.28	0.27	0.27	0.28	0.28
Israel Jewish majority	Middle childhood	0.26	0.34	0.34	0.31	0.35	0.35
Israel Jewish majority	Late childhood	0.25	0.35	0.35	0.33	0.36	0.36
Israel Jewish majority	Adolescence	0.19	0.26	0.25	0.25	0.26	0.26
Italy	Late childhood	0.17	0.29	0.28	0.26	0.29	0.29
Poland	Middle childhood	0.14	0.18	0.18	0.18	0.18	0.18
Poland	Late childhood	0.21	0.29	0.29	0.28	0.30	0.30
Poland	Adolescence	0.16	0.25	0.25	0.23	0.25	0.25
Portugal	Late childhood	0.34	0.41	0.41	0.40	0.42	0.42
Switzerland	Middle childhood	0.28	0.33	0.32	0.31	0.33	0.33
Switzerland	Late childhood	0.22	0.34	0.34	0.33	0.35	0.35

In the normative model, the circle of values, including the major conflicts among the four higher-order values, was largely replicated across samples. The organization of values within this structure varied somewhat by sample, with some samples showing clearer organization than others. For example, in some samples, values of self-transcendence and conservation were intermixed and not clearly distinguished. In addition, in a number of samples, power values were strongly distinguished from other values. Importantly, deviance from the structure was not likely to include proximity of conflicting values or lack of value differentiation. Of the 260 possibilities for a deviation of a value into a neighboring area in the value circle (10 items * 2 neighboring area * 13 samples), only two deviations were found for benevolence values and two for achievement values (1%). Of the 130 possibilities for a deviation of a value into a conflicting area (10 items * 1 conflicting area across the circle * 13 samples), only one case was found. Specifically, self-direction values in the middle childhood sample in Switzerland were located within the conflicting conservation values area (1%).

The distribution of misfit of individuals to the structure is summarized in Table 4. Misfit was lower in older samples, as calculated by weighted means and *SD*s. Further details of the unfolding solutions by sample are presented in the supplemental material, including the joint configuration plots (SM 2), charts showing contribution of values and individuals to stress (SM 3), and a table summarizing contribution of values to stress (SM 4).

**Table 4.** *K* Distribution indicating the extent of divergence of individuals from the value structures.

Country	Age group	Normative model		Theoretical model	
		*Mean*	*SD*	*Mean*	*SD*
Australia	Middle childhood	0.17	0.06	0.18	0.05
Australia	Late childhood	0.18	0.06	0.23	0.06
Israel Palestinian citizens	Adolescence	0.19	0.07	0.25	0.08
Israel Jewish majority	Middle childhood	0.25	0.07	0.30	0.06
Israel Jewish majority	Late childhood	0.24	0.07	0.32	0.07
Israel Jewish majority	Adolescence	0.18	0.07	0.23	0.07
Italy	Late childhood	0.16	0.05	0.25	0.07
Poland	Middle childhood	0.13	0.05	0.16	0.06
Poland	Late childhood	0.20	0.07	0.27	0.08
Poland	Adolescence	0.15	0.05	0.22	0.06
Portugal	Late childhood	0.32	0.08	0.40	0.06
Switzerland	Middle childhood	0.27	0.06	0.30	0.06
Switzerland	Late childhood	0.20	0.08	0.31	0.08
Overall	Middle childhood	0.22	0.06	0.27	0.06
	Late childhood	0.20	0.06	0.27	0.06
	Adolescence	0.16	0.06	0.23	0.06

Linear latent growth curve modeling was used to estimate the change in value importance over time in samples with more than three time points. Of the 90 estimated models (10 values * 9 samples), 76 (84%) fit the data excellently, and 89 (99%) adequately, on at least one fit index (SM 5). The absolute maximal change and variance in change (in *s* and *d*) across values in each sample are presented in Tables 5 and 6, respectively.

**Table 5.** Distribution of *s*, as an Indicator of individual-level change in values over time, resulting from the latent growth curves.

Country	Age group	Distribution of slope variances				Distribution of maximum absolute slope values			
		*Mean*	*SD*	*Min*	*Max*	*Mean*	*SD*	*Min*	*Max*
Israel Palestinian citizens	Adolescence	0.01	0.01	0.00	0.06	0.18	0.09	0.05	0.74
Israel Jewish majority	Middle childhood	0.03	0.03	0.00	0.15	0.35	0.18	0.09	1.01
Israel Jewish majority	Adolescence	0.004	0.003	0.00	0.02	0.11	0.05	0.03	0.33
Italy	Late childhood	0.002	0.002	0.00	0.02	0.09	0.04	0.05	0.41
Poland	Middle childhood	0.003	0.003	0.00	0.04	0.10	0.05	0.02	0.32
Poland	Late childhood	0.03	0.01	0.00	0.10	0.31	0.11	0.08	0.78
Poland	Adolescence	0.04	0.04	0.01	0.37	0.36	0.19	0.15	1.33
Switzerland	Middle childhood	0.01	0.01	0.00	0.07	0.19	0.07	0.06	0.43
Switzerland	Late childhood	0.02	0.01	0.01	0.08	0.25	0.07	0.12	0.63

**Table 6.** Distribution of *d*, as an indicator of individual-level change in values over time, resulting from the difference test.

Country	Age group	Variance of difference				Maximum absolute difference			
		*Mean*	*SD*	*Min*	*Max*	*Mean*	*SD*	*Min*	*Max*
Australia	Middle childhood	0.74	0.45	0.06	2.66	1.63	0.59	0.45	3.45
Australia	Late childhood	0.64	0.40	0.08	2.74	1.53	0.57	0.45	3.75
Israel Palestinian citizens	Adolescence	0.88	0.48	0.11	2.67	1.63	0.54	0.50	3.50
Israel Jewish majority	Middle childhood	0.81	0.65	0.05	5.51	1.61	0.71	0.33	5.53
Israel Jewish majority	Late childhood	0.67	0.46	0.06	2.46	1.46	0.56	0.43	3.50
Israel Jewish majority	Adolescence	0.63	0.54	0.08	4.03	1.37	0.60	0.45	4.03
Italy	Late childhood	0.32	0.38	0.00	2.78	1.00	0.59	0.00	4.30
Poland	Middle childhood	0.45	0.38	0.04	5.32	1.20	0.51	0.26	5.32
Poland	Late childhood	1.60	0.90	0.00	4.67	2.27	0.86	0.00	4.67
Poland	Adolescence	0.78	0.43	0.06	2.56	1.54	0.53	0.50	3.50
Portugal	Late childhood	1.14	1.26	0.03	9.44	1.90	1.16	0.40	9.44
Switzerland	Middle childhood	0.94	0.84	0.00	6.47	1.71	0.82	0.00	6.47
Switzerland	Late childhood	0.78	0.55	0.11	2.89	1.57	0.64	0.50	3.50

### Associations between value structure organization and value change

**Figure 3.**
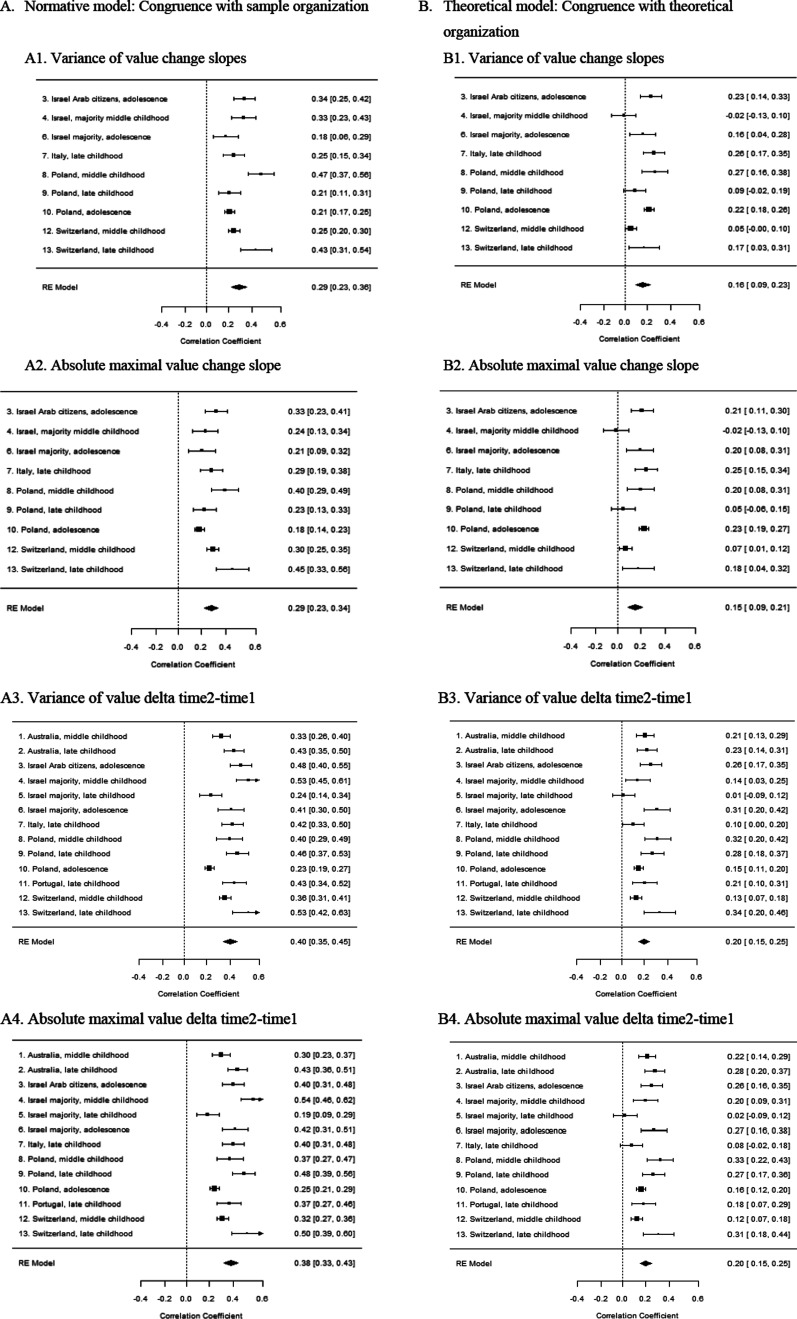
Meta-analysis summary forest plots: (a) Normative model: congruence with sample organization; (b) theoretical model: congruence with theoretical organization. *Note*. The polygon at the bottom of each forest plot represents the summary effect size, with the width of the polygon representing the 95% confidence interval. A point estimate represents each sample, bounded by the effect CI. The size of box for each study represents the study contribution to the summary effect size.

The random-effects meta-analysis across samples indicated that the vast majority of weighted mean effect sizes for the associations between fit and value change were positive and significant, indicating that children whose values at the first time point are less congruent with the structure of values are more likely to change their value priorities over time. These results, including the effect sizes and *CI*s for each sample, along with the computed summary effect sizes, are visualized in forest plots in Figure 3. Specifically, of the 88 effect sizes computed between incongruence with structure and value change (normative/theoretical structure (2) *s/*d* (2) * absolute maximal/variance (2) * sample number (*K* = 9/13)), 80 were positive and significant (91%), 6 were positive but not significant, and only 2 were negative and not significant (Figure 3). The pooled associations between misfit and *s* indicators (the latent linear slope of value change across time for 9 samples with more than three time points) ranged between *r* = .15 and *r* = .29. The associations between misfit and *d* indicators for all samples ranged between *r* = .20 and *r* = .40. This supports the proposition that incongruence in personality can be associated with personality change over time.

The associations between value change and incongruence with the structure appeared stronger and more consistent for the normative model, reflecting value organization of the sample (pooled *r* ranging between .29 and .40), than for the theoretical model, reflecting the theoretical structure of values (pooled *r* ranging between .15 and .20). To understand the magnitude of this difference, we compared the normative and theoretical pooled effect 95% confidence interval within each index type of model (comparing slopes and *d*s, variance and absolute maximum). In all four comparisons, the differences were significant, suggesting that the estimated effects differ.

Table 7 displays tests of heterogeneity in the effect sizes across samples, demonstrating that most of the observed variation can be attributed to differences between samples, rather than within samples. The significant *Q* statistic indicates that the true effect is different across samples and cannot be attributed merely to chance. Interestingly, *Q*s are higher in the normative model relative to the theoretical model, indicating higher heterogeneity between samples in the normative model. Similarly, the *I*^2^ and *Tau*^2^ statistics indicate that a high to moderate proportion of the observed variation in both the normative and theoretical models can be attributed to differences between samples, rather than within-sample variation. These results indicate that in different cultures and age groups, fit to the value structure had different associations with value change.

**Table 7.** Heterogeneity of effects across studies.

	*Q*			*I* ^*2*^ *(%)*	*Tau* ^*2*^
	*Estimate*	*df*	*p*		
*Free model*					
Variance of slopes	36.04	8	<.001	82.97	0.01
Absolute maximal slope	33.32	8	<.001	74.89	0.01
Variance of delta	92.13	12	<.001	84.23	0.01
Absolute maximal slope	80.20	12	<.001	85.01	0.01
*Restricted model*					
Variance of slopes	44.48	8	<.001	80.93	0.01
Absolute maximal slope	41.18	8	<.001	78.65	0.01
Variance of delta	42.98	12	<.001	76.62	0.01
Absolute maximal slope	43.67	12	<.001	76.29	0.01

Finally, as shown in Table 8, the results of a meta-regression analysis investigating the role of age group in accounting for the study heterogeneity showed very little role for age in the moderation of the associations between value change and congruence with the structure. One significant comparison indicated that adolescents were more likely to have a positive association between value change and incongruence with the structure than children in middle childhood. However, this is only one significant association among 16 comparisons. Thus, the results suggest a common process across age groups.

**Table 8.** Moderation meta-analysis models.

	Age group	*b*	*CI*	*se*	*Z*	*p*
*Free model*						
Variance of slopes	Intercept	0.361	[0.232, 0.490]	0.066	5.477	.000
	Late versus middle	−0.060	[−0.246, 0.126]	0.095	−0.634	.526
	Adolescence versus middle	−0.113	[−0.295, 0.068]	0.092	−1.226	.220
Absolute maximal slope	Intercept	0.320	[0.216, 0.424]	0.053	6.021	.000
	Late versus middle	0.007	[−0.145, 0.158]	0.077	0.087	.931
	Adolescence versus middle	−0.079	[−0.225, 0.067]	0.074	−1.064	.287
Variance of delta	Intercept	0.428	[0.310, 0.547]	0.060	7.107	.000
	Late versus middle	0.018	[−0.137, 0.172]	0.079	0.225	.822
	Adolescence versus middle	−0.041	[−0.221, 0.139]	0.092	−0.446	.655
Absolute maximal delta	Intercept	0.404	[0.282, 0.527]	0.062	6.491	.000
	Late versus middle	0.017	[−0.142, 0.176]	0.081	0.208	.835
	Adolescence versus middle	−0.038	[−0.224, 0.148]	0.095	−0.403	.687
*Restricted model*						
Variance of slopes	Intercept	0.097	[−0.015, 0.208]	0.057	1.692	.091
	Late versus middle	0.081	[−0.081, 0.244]	0.083	0.982	.326
	Adolescence versus middle	0.114	[−0.043, 0.270]	0.080	1.420	.156
Absolute maximal slope	Intercept	0.080	[−0.012, 0.173]	0.047	1.698	.090
	Late versus middle	0.080	[−0.057, 0.216]	0.070	1.144	.253
	Adolescence versus middle	0.139	[0.009, 0.268]	0.066	2.100	.036
Variance of delta	Intercept	0.199	[0.099, 0.299]	0.051	3.896	.000
	Late versus middle	−0.005	[−0.137, 0.126]	0.067	−0.080	.936
	Adolescence versus middle	0.041	[−0.111, 0.193]	0.078	0.528	.598
Absolute maximal delta	Intercept	0.214	[0.114, 0.314]	0.051	4.208	.000
	Late versus middle	−0.023	[−0.153, 0.108]	0.067	−0.337	.736
	Adolescence versus middle	0.014	[−0.137, 0.166]	0.077	0.183	.855

## Discussion

For the first time, we found that incoherence with the value structure predicts value change over time. The investigation was conducted in 13 longitudinal samples of children and adolescents, from seven cultures in six countries. Importantly, we suggest a robust, theory-based, and methodologically sophisticated approach to test the idea that incoherence in personality is associated with later personality change (Quirin & Kuhl, 2022). In this study, we test the role of personality incoherence in predicting change, for the first time in an entire system of a central aspect of personality (in this case, personal values). Although tested with values, the results suggest that similar processes may take place in other personality aspects, such as narrative identity and traits. These processes, however, are more difficult to test as they do not include a clear operationalization of internal conflict.

### Value coherence and value change over time

In the vast majority of samples, the value structure was already quite coherent at Time 1, as documented by the *Stress I* of values, which indicated that the theoretical structure of values described children’s value priorities well. The normative structure for each sample largely replicated the theoretical structure of values but allowed for some variation. Our results coalesce with previous studies, in finding that the structure of values is rather coherent, yet further develops (Daniel et al., 2020, 2023), as children become more likely to distinguish specific basic values, in contrast to higher-order value dimensions (Abramson et al., 2018; Lee et al., 2017).

Results indicated that children and adolescents whose reported values were less coherent at the first time point were more likely to show change in their values over time. These results were highly robust across samples. They were also robust across types of value change. Specifically, children who had a less coherent value system were more likely to report multiple values that changed in multiple directions (as indicated by the variance of value index) and one value that changed drastically (as indicated by the absolute maximum value index). They were also more likely to show value change between two waves of data collection, as well as long-term change across multiple waves of data collection. These results provide strong evidence that incoherence in the structure of children’s values is an indicator of personality incoherence that precedes change.

Although the results convincingly document an association between value incoherence and later value change, they do not offer a mechanism that may explain it. A number of such mechanisms may contribute to the process. Multiple theories suggest that individuals hold an *internal* drive to seek self-coherence (e.g., Dweck, 2017). In the absence of self-coherence, individuals feel psychologically unrooted or lacking in self-integrity. Thus, value change may be an attempt to ameliorate tension created by lack of coherence. Further, value incoherence may hinder the role of values in driving behavior. If individuals value two conflicting goals to a similar extent they must find a solution to allow them to pursue any of the goals (Kung & Scholer, 2020). Other theories suggest an external drive to seek social coherence. Individuals internalize values from their social and cultural groups (Daniel et al., 2012). If the social group embraces a coherent value-system, increased acceptance of its values may lead group members to a more coherent value system. Future studies may use the newly developed methodology demonstrated in this study to test these possible mechanisms.

Regardless of the mechanism, our overall finding supports theoretical concepts of increase in personality coherence as a process of self-growth, in which individuals successfully integrate personal experiences into a coherent network (Quirin & Kuhl, 2022). Values were a particularly good candidate to test this proposition, as they are organized coherently and consistently, providing a clear marker to identify a coherent personality network. Unlike values, the main personality trait model, the Big Five model (e.g., John & Srivastava, 1999), and its relatives (e.g., HEXACO, Ashton & Lee, 2007) have an organization of specific traits subsumed under more general traits, with the more general traits often organized as quite orthogonal to one another. Orthogonality makes it difficult to specify what relations among traits are not likely to lead to positive outcomes, as each pair of broad traits can co-exist. There is also no theory that specifies that certain trait combinations are more difficult to have, apart from the contents of certain traits being conducive to negative personal outcomes (especially high neuroticism, e.g., Steel et al., 2008). Hence, while it is possible to test personality coherence in terms of having the same traits across contexts, it is not possible to test it in terms of the internal organization of traits.

There is currently no evidence for the association between value structure coherence and well-being. Nevertheless, there is evidence for a positive association between value coherence across contexts in one’s life and well-being (Daniel, Boehnke, & Knafo-Noam, 2016). Similarly, research shows positive associations between well-being and coherence between one’s values and the values of one’s social environment. These associations were found in the case of the national social environment (Hanel et al., 2020; Wolf et al., 2021), the community (Sortheix et al., 2013), fellow students (Sortheix & Lönnqvist, 2015), classmates (Benish-Weisman et al., 2020), and romantic partners (Leikas et al., 2018). Future research could examine whether value structure incoherence is associated with well-being.

Value change processes adhere to the value structure (Bardi et al., 2009; Daniel & Benish-Weisman, 2019). As individuals change in the importance of one value, they are also likely to change in the importance of conflicting values in the opposite direction. For example, immigrants who increase the importance they ascribe to self-direction values over time are likely to decrease the importance they ascribe to the opposing conformity values (Bardi et al., 2009, 2014). Similarly, experimental studies found that priming one value causes a decrease in the importance of opposing values (Maio et al., 2009). The current results suggest that the process of value change may progress over time. As individuals increase in the importance of one value, their value coherence may be compromised, leading to further change in other values in order to restore coherence. Thus, the value incoherence identified here may not only be an antecedent of change but also its consequence.

### Value change in childhood and adolescence

The hypothesis that incoherence in value structure precedes value change was investigated across time and in different age groups, between middle childhood and adolescence. Previous studies indicated evolvement in value structure during middle childhood. In previous studies (Abramson et al., 2018; Cieciuch et al., 2016; Daniel et al., 2023), and in samples in the current study, children reported a relatively coherent structure of values. Nevertheless, children in middle childhood were more likely than those in late childhood to report a less differentiated value system, in which all values were moderately important. Their values were also likely to become more differentiated over time, reflected in patterns of value priorities that adhere to the basic principles of the value structure (Daniel et al., 2020). Moreover, longitudinally and across cultures, children were more likely to report a coherent value system with age, especially between 6 and 10 years of age (Daniel et al., 2023). In the current study as well, younger samples showed less coherent value systems than older samples. Thus, incoherence in the value structure may reflect processes of maturation as the value structure develops with age. The current investigation also provides evidence that maturation with age is likely to be accompanied by changes in value importance. However, we found that age had little effect on the associations between value change and congruence with the structure.

The change in the structure of values in youth accompanies changes in value priorities. During adulthood, values are considered to be stable individual characteristics. Individuals are likely to maintain their value priorities over years, with changes being mostly temporary, or in the face of major changes in their environment (Daniel et al., 2022; Schuster et al., 2019). In contrast, both children and adolescents show changes in their value priorities over the years (Cieciuch et al., 2016; Daniel & Benish-Weisman, 2019; Daniel et al., 2020; Vecchione et al., 2020). This fluidity in value priorities may be a marker of a lack of maturity in value importance. Theory suggests values change with age as a result of changing social demands and social environments in which children function (Bardi & Goodwin, 2011; Döring et al., 2016). The current study suggests that it may also result from immaturity reflected in the incoherence in the value systems of children, furthering an exploration of values until reaching a coherent, and stable, value system.

Interestingly, the current study did not identify a moderating effect of age. Thus, individuals who held less coherent value systems in middle-childhood, late childhood, and adolescence were equally likely to report changes in their values over time. Some previous theories focused on adolescence as the period of identity formation, in which adolescents explore different value options, weight them, and decide upon the values they adopt and maintain (Erikson, 1968; Meeus, 2011). Our results suggest that this exploration may already be present during middle childhood, although it is not clear whether such exploration is intentional. Moreover, previous studies suggest that children and adolescents gradually become more adept at identifying their internal conflicts and become more adept in tolerating such conflicts with little discomfort (Daniel, Boehnke, & Knafo-Noam, 2016; Daniel et al., 2012; Harter, 2012; Harter & Monsour, 1992). Thus, although children may change in the structure of their values, cognitive advances may make value incoherence carry different meaning across ages.

### Cross cultural aspects of value change

The most consistent result in the current investigation is the robust associations across samples. Thus, it appears that the process described here (of change following incoherence) is not culture specific. Past studies of value development, investigating changes in value importance across ages, and changes in value coherence across ages, found indications for parallel processes across cultures (Daniel & Benish-Weisman, 2019; Daniel et al., 2012, 2023). Our study goes a step further, to show that value change follows incoherence across both age and cultural groupings. Nevertheless, additional research is required in order to investigate our initial conclusion further and to understand its boundaries. Such work could try to account for the heterogeneity in effects that were demonstrated, but not explained, in the meta-analysis.

Importantly, the current investigation is constricted in the nature of the cultural groups it covers. It has a strong bias towards Western cultures, despite including some exceptions. This is important, as the very conceptualization of personality coherence may vary across cultures (Fajkowska, 2022). Countries characterized by dialectical thinking, see contradiction as a fact of life to be accepted, and not a logical problem to solve. Their preferred approach to an apparent contradiction is not choice among options but compromise (de Oliveira & Nisbett, 2017; Peng & Nisbett, 1999). Thus, if incoherence promotes change by creating psychological unease, the effects may be different in cultures promoting dialectical thinking. In contrast, if incoherence promotes change by making value-fulfillment more difficult in the presence of competing goals, or through other mechanisms, the process identified here is likely to be similar across these cultures. Future studies should widen the cultural coverage of the current investigation to new cultures.

### Standards of comparison: Age and culture specificity

The current study investigated the coherence with a value structure that is specific to the sample (normative), as well as with the theoretical structure of values as hypothesized by Schwartz (1992). The results are consistent across both structures, yet coherence with the sample’s normative structure was more strongly associated with value change. Hence, deviation from the normative value structure in one’s particular age group and culture may drive value change to a greater extent than deviation from the theoretical structure.

The development of the value structure with age could suggest that the normative (sample-driven) value structure may not be an appropriate standard to compare the value structure of individual children. The normative structure can be interpreted to represent a meaningful system of inter-relations that is unique and characteristic of a specific context. Alternatively, it can be interpreted as an error-random deviation from the theoretical structure. The replication of our results across the two reference structures supports the validity of the normative structure as a reference point. Moreover, as previously discussed, in all our samples, and in previous child and adolescent studies (e.g., Döring et al., 2015), the theoretical structure of values was replicated, with some minor deviations.

Further, the value structure of children and adolescents may not only reflect immaturity-related deviations from adult samples but also normative effects. The normative structure of values can arise from sample-specific meaning of values. For example, in our study, many of the normative structures showed achievement values closer to conservation, rather than power values, possibly revealing age- or culture-related meaning of achievement values. For instance, education systems may conflate achievement and conformity, by evaluating students based on their obedience or imposing rules to regulate academic investment and aspiration. This may lead children to understand achievement and conformity values as being more interrelated than adults do. Alternatively, the results may suggest that the measurement of achievement values in children’s instruments should be further explored. In both cases, this is an example of a situation in which the comparison to age-specific norms may reflect an underlying meaning relevant to the specific age and/or cultural group.

### Strengths, limitations, and future directions

Our study has several strengths. First, it applied a well-organized and comprehensive value theory that has been validated across cultures (Fontaine et al., 2008; Sagiv & Schwartz, 2022) and age groups (Twito-Weingarten &amp; Knafo-Noam, 2022Twito-Weingarten & Knafo‐Noam, 2022). This enabled the current study to overcome past difficulties in the investigation of coherence in personality by testing the effect of incoherence with the structure of values on value change. Second, our research included a substantial number of samples of children and adolescents that varied in culture, age-group, length of time between measurements, measures, procedures, and more. Despite this variability, the effects were robust across samples. Thus, this study provides a comprehensive investigation of the research questions. Third, this study examined development by following the same children over time, in longitudinal samples. This state-of-the-art design overcomes cohort effects and can identify within-individual processes.

This study also has several limitations. First, we relied on values as reported by participants. Self-report measures can be biased, suffering from social desirability, among other limitations. However, self-report is the most effective measure of value importance to date. Moreover, social desirability is not a bias in value self-reports but an important trait that is meaningfully associated with value importance (Schwartz et al., 1997). A second limitation is the use of different measures to assess values across ages. Childhood samples applied visual and verbal measures (PBVS-C or AVI-r), while adolescent samples applied only verbal measures (PVQ40 and PVQ-RR 57). These differences arise from the very nature of developmental research, as studies applied measures appropriate for participants’ cognitive skills. However, in each age-group, at least two different measures were used, making the robust effects found in the current study independent of the measures used. Third, the current samples suffered from attrition, a common problem in longitudinal studies. While calculation of slope of change accounted for missing values using the Maximum Likelihood algorithm (Mirzaei et al., 2022), the unfolding analysis included only participants who completed T1, and the *d* index included only participants who completed T1 and T2. Thus, we could not correct against bias due to missingness in this analysis. Finally, as previously mentioned, the cultures sampled were restricted to those in which children’s and adolescent’s values have been studied longitudinally. While Western samples were overrepresented, the samples included differed along important cultural characteristics. For instance, based on Hofstede’s cultural dimensions, Australia is high on individualism (90) in contrast to Portugal (27), Poland is high on power distance (68) in contrast to Israel (13), Italy is high on masculinity (70) in contrast to Portugal (31), and Portugal is high on uncertainty avoidance (104) in contrast to Australia (51; Hofstede, 2023).

Our investigation focused on incoherence in the value system as a predictor of change. The conceptual work on personality incoherence suggests lower well-being is a consequence of incoherence in personality (Quirin & Kuhl, 2022). Future research may use our measure of value incoherence to test this claim. One can also apply our procedure to test whether a coherent value system predicts stronger associations between values and related constructs, such as goals, attitudes, and identities, contributing further to a highly coherent integrative self.

### Conclusions and implications

Our results support existing theories regarding the role of coherence within personality in personality development (Fournier et al., 2015; Quirin & Kuhl, 2022), by testing them within a well-validated, comprehensive system of a central personality aspect. We demonstrate that children and adolescents who prioritize conflicting values similarly were more likely to report different values over subsequent measurement points. Put differently, disorganization in one’s motivational self may be associated with reorganization of one’s value priorities. The results carry weight for future interventions in value importance. They suggest that an intervention in the importance of one value may carry further changes in additional values, to resume integration and coherence of the value system.

## Supplemental Material

Supplemental Material - Value incoherence precedes value change: Evidence from value development in childhood and adolescence across cultures Supplemental Material for Value incoherence precedes value change: Evidence from value development in childhood and adolescence across cultures by Ella Daniel, Anat Bardi, Julie Lee, Ricarda Scholz-Kuhn, Einat Elizarov, Jan Cieciuch, Ariel Knafo-Noam, Alice Ramos, Michele Vecchione, Rene Algesheimer, Evelia Murcia Alvarez, Avital Ben Dror Lankry, Maya Benish-Weisman, Ricardo Borges Rodrigues, Anat Chomsky, Patricia Collins, Eldad Davidov, Anna K Döring, Stefanie Habermann, Dana Katsoty, Martin Kindschi, Elena Makarova, Gilda Marsicano, Kinneret Misgav, Thomas P Oeschger, Leonor Pereira da Costa, Joanne Sneddon, Iva Tendais, and Louise Twito-Weingarten in European Journal of Personality.
