# Supplementary material for: Value incoherence precedes value change: Evidence from value development in childhood and adolescence across cultures
Source: Eur J Pers. 2024 Nov 19;39(5):677–96. doi: 10.1177/08902070241289969 (PMC13038160; doi:10.1177/08902070241289969)
Supplement: Supplemental Material - Value incoherence precedes value change: Evidence from value development in childhood and adolescence across cultures [file sj-pdf-1-erp-10.1177_08902070241289969.pdf]

Value Incoherence Precedes Value Change: Evidence from Value Development in Childhood and Adolescence Across Cultures

Supplemental Material

Supplemental Material (SM) 1. Sample items.

Value	PVQ40/PVQ-RR 57 (respectively, male version)	PBVS-C		AVI-r
Benevolence	It's very important to him to help the people around them. He wants to care for their well-being.<br><br>It is very important to him to help the people dear to him.	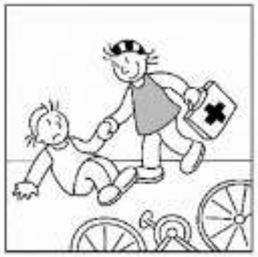	to help others	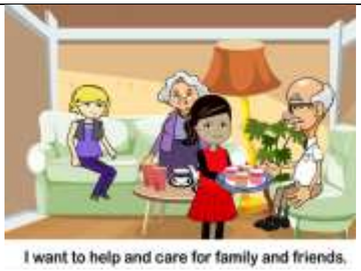
Power	It is important to them to be rich. They want to have a lot of money and expensive things.<br><br>It is important to him to have the power that money can bring.	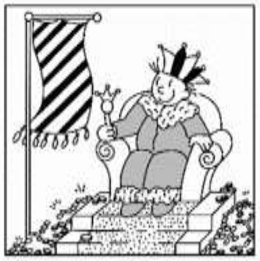	to be rich and powerful	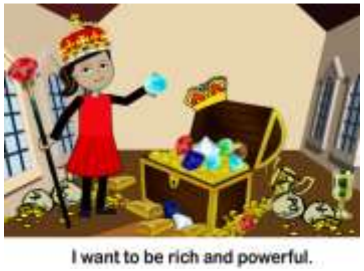

Hedonism	Having a good time is important to them. They like to "spoil" themselves.<br><br>It is important to him to enjoy life's pleasures.	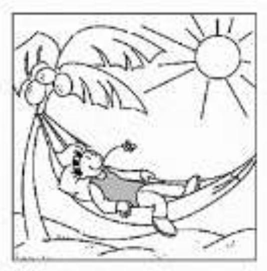	to enjoy life	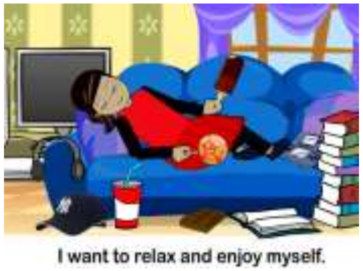
Security	It is important to them to live in secure surroundings. They avoid anything that might endanger their safety.<br><br>It is important to him to be personally safe and secure.	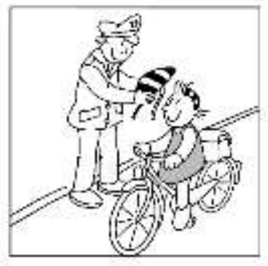	to be safe	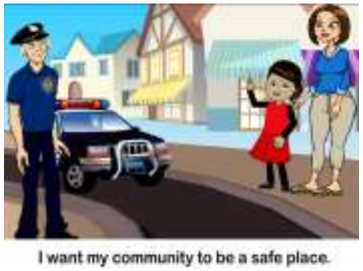

Supplemental Material (SM) 2. Unfolding solutions by sample: Joint configuration plots

Country	Age group	Unfolding free	Unfolding restricted
Australia	Middle childhood	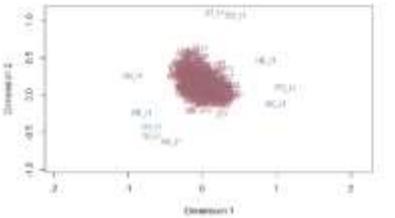	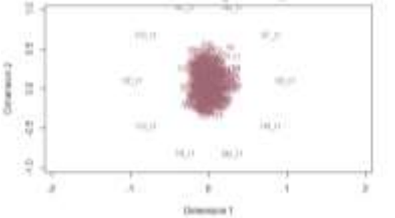
Australia	Late childhood	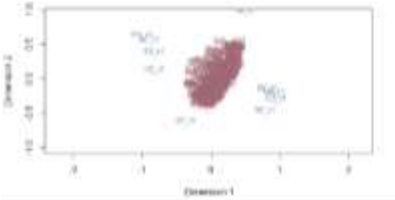	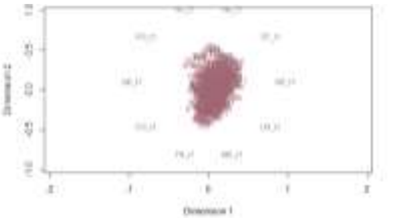
Israel Arab citizens	Adolescence	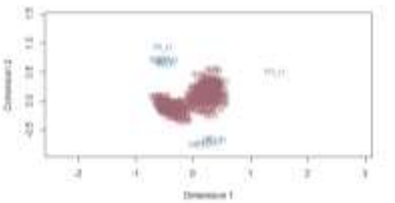	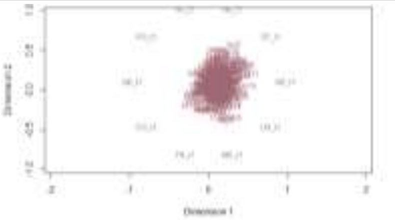
Israel Jewish majority	Middle childhood	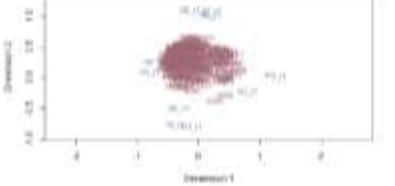	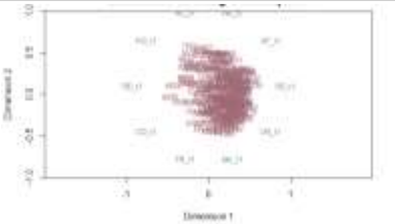
Israel Jewish majority	Late childhood	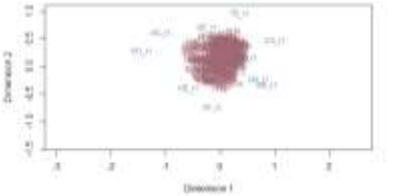	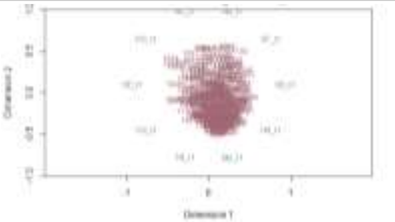
Israel Jewish majority	Adolescence	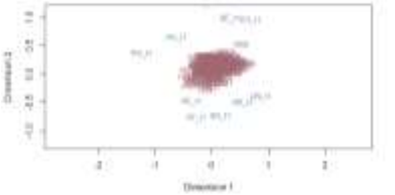	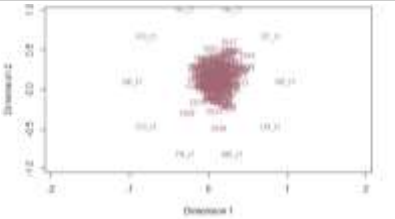

Italy	Late childhood	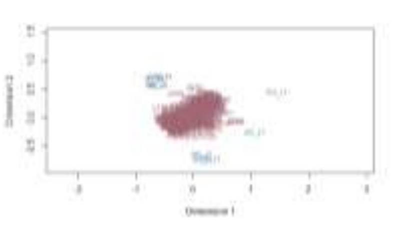	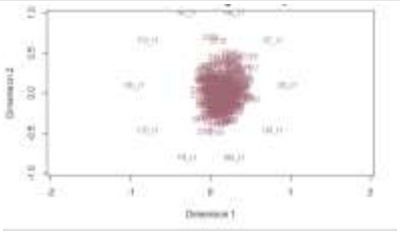
Poland	Middle childhood	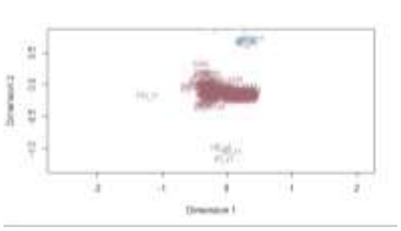	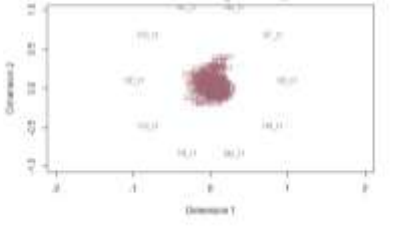
Poland	Late childhood	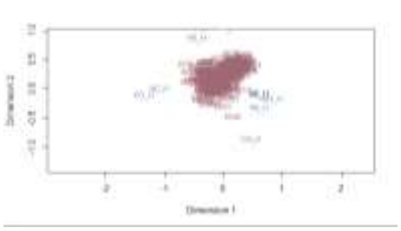	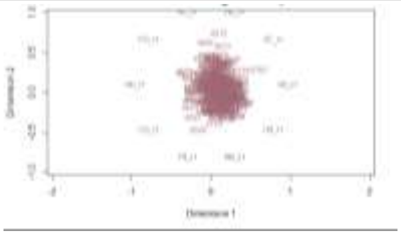
Poland	Adolescence	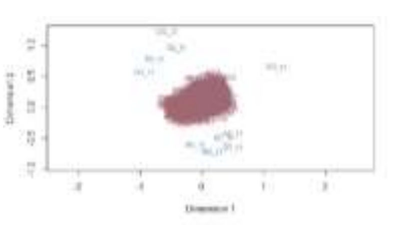	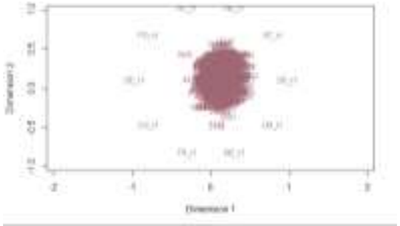
Portugal	Late childhood	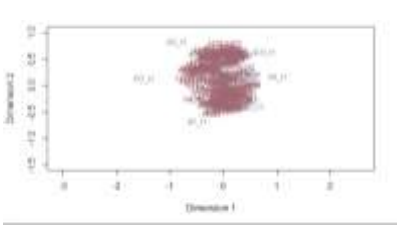	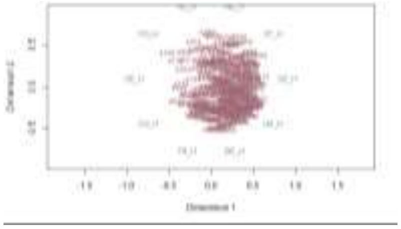
Switzerland	Middle childhood	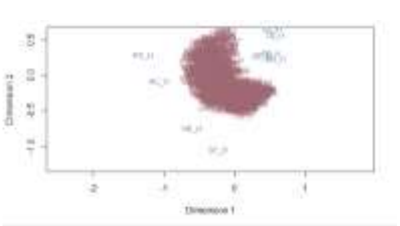	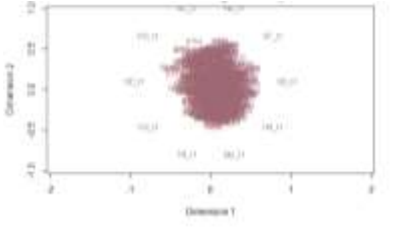
Switzerland	Late childhood	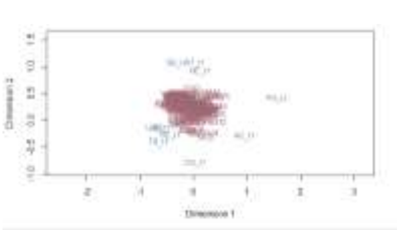	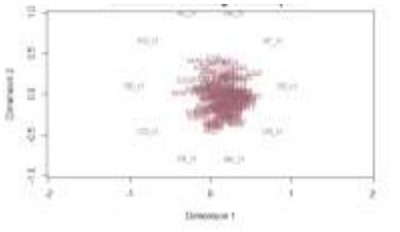

Supplemental Material (SM) 3. Contribution of values and individuals to stress

Country	Age group	Unfolding free	Unfolding restricted
Australia	Middle childhood	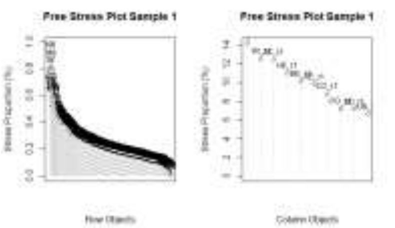	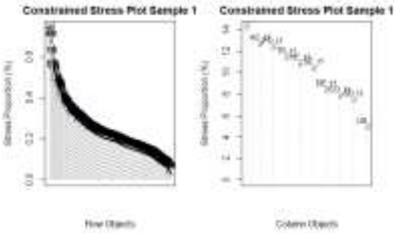
Australia	Late childhood	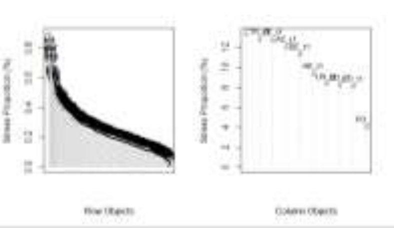	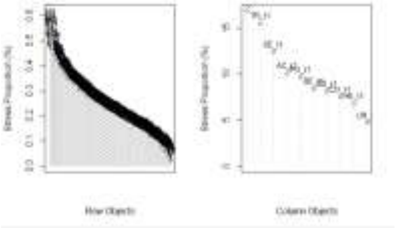
Israel Arab citizens	Adolescence	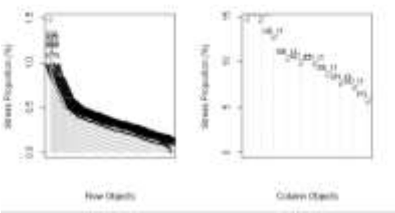	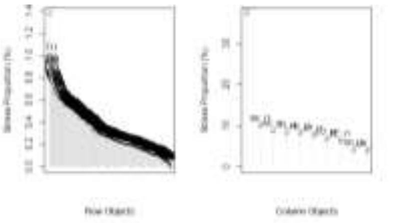
Israel Jewish majority	Middle childhood	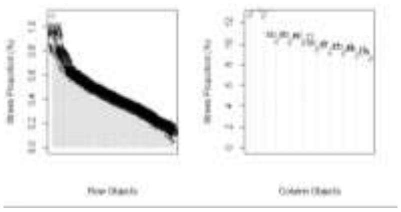	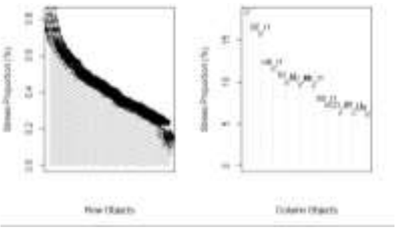
Israel Jewish majority	Late childhood	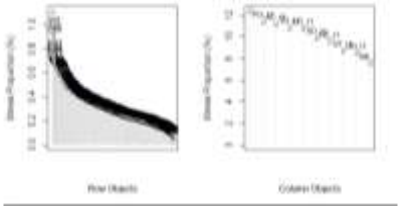	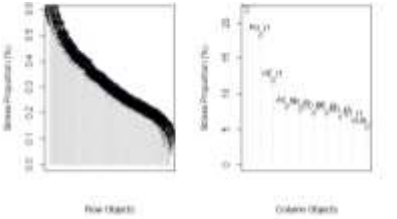

Israel Jewish majority	Adolescence	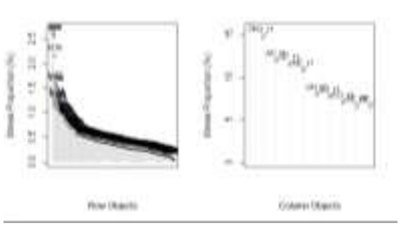	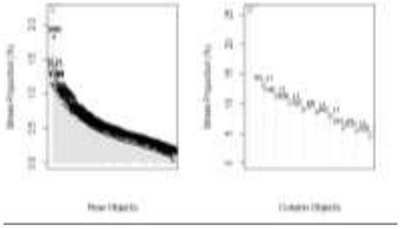
Italy	Late childhood	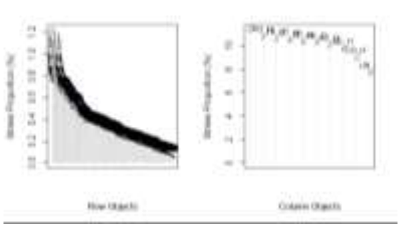	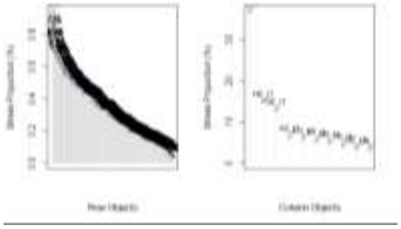
Poland	Middle childhood	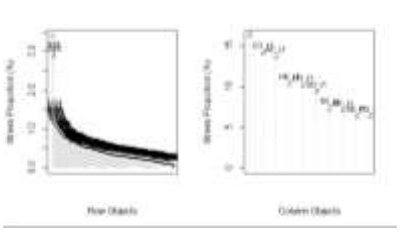	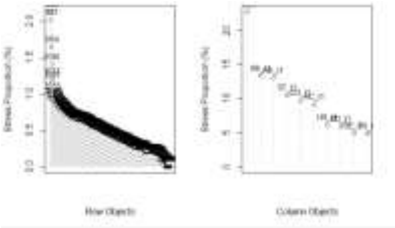
Poland	Late childhood	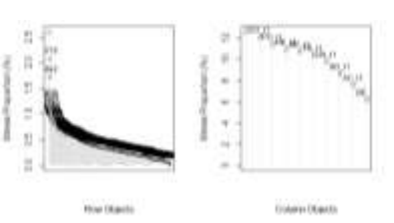	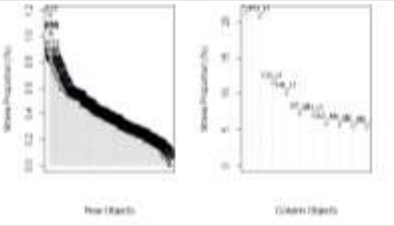
Poland	Adolescence	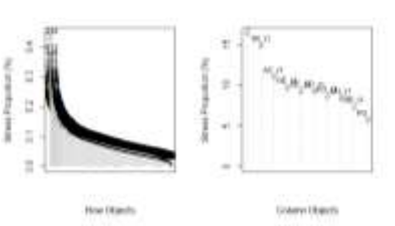	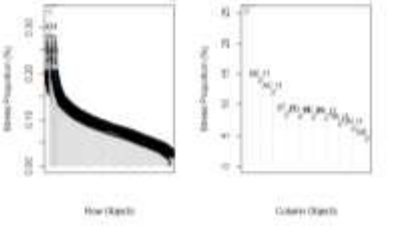
Portugal	Late childhood	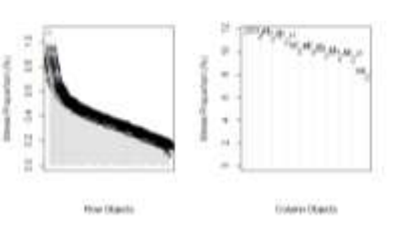	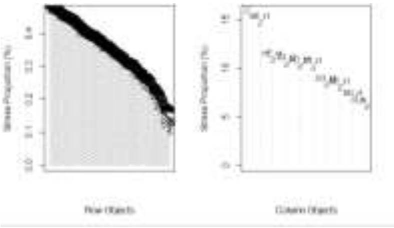

Switzerland	Middle childhood	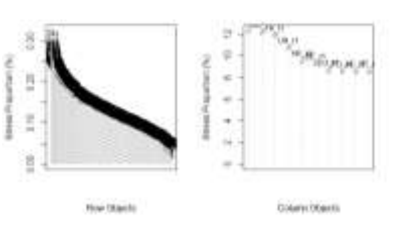	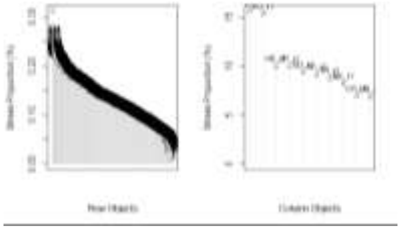
Switzerland	Late childhood	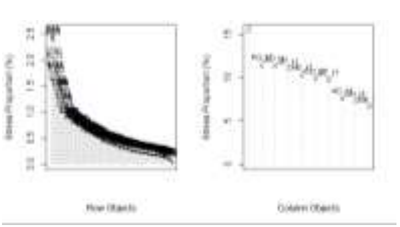	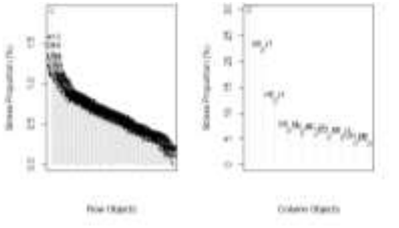

Supplemental Material (SM) 4. Contribution of values to the unfolding solution stress

Country	Age group	Self-direction	Stimulation	Hedonism	Achievement	Power	Security	Conformity	Tradition	Benevolence	Universalism
Free											
Australia	Middle childhood	7.14	14.22	11.02	12.49	7.19	9.86	8.79	12.52	10.15	6.62
Australia	Late childhood	8.20	13.37	9.34	12.01	3.98	12.72	8.03	12.78	11.28	8.29
Israel Arab citizens	Adolescence	7.04	14.65	12.57	9.77	5.55	8.50	9.67	14.46	10.25	7.54
Israel Jewish majority	Middle childhood	10.12	9.09	12.60	9.99	12.67	9.32	8.97	10.04	8.75	8.44
Israel Jewish majority	Late childhood	9.83	8.61	9.47	11.14	11.32	12.33	10.70	10.75	7.44	8.43
Israel Jewish majority	Adolescence	7.66	11.43	10.75	11.91	14.70	6.52	6.99	15.62	6.51	7.91
Italy	Late childhood	11.37	10.44	10.23	10.03	10.73	9.72	8.95	10.55	10.30	7.66
Poland	Middle childhood	9.29	16.19	10.21	13.57	6.22	6.24	14.06	7.14	6.98	10.09
Poland	Late childhood	8.62	12.60	10.88	7.55	11.40	10.69	12.06	10.32	6.15	9.73
Poland	Adolescence	9.17	16.44	9.63	10.96	5.63	9.19	8.59	14.81	7.25	8.32
Portugal	Late childhood	9.55	10.75	9.82	9.16	11.01	9.88	11.21	11.69	7.62	9.30
Switzerland	Middle childhood	12.32	8.51	9.50	8.53	8.54	12.16	8.61	11.87	9.27	10.70
Switzerland	Late childhood	11.28	10.92	10.24	7.56	11.36	9.66	9.82	15.39	6.55	7.22
Constrained											
Australia	Middle childhood	7.38	14.29	10.78	12.63	12.31	10.36	7.76	11.36	8.33	4.79

Australia	Late childhood	8.12	16.91	6.76	10.08	9.66	12.40	7.48	15.54	8.37	4.68
Israel Arab citizens	Adolescence	4.01	7.48	8.03	8.67	37.34	9.85	6.61	8.21	6.13	3.68
Israel Jewish majority	Middle childhood	7.13	6.29	11.49	9.55	18.25	15.66	6.29	9.85	9.50	5.99
Israel Jewish majority	Late childhood	6.78	6.12	11.80	8.07	18.23	21.66	7.25	7.72	7.24	5.13
Israel Jewish majority	Adolescence	4.43	8.43	11.07	7.68	25.65	8.81	5.30	12.92	9.94	5.78
Italy	Late childhood	5.19	5.59	15.02	6.48	36.99	13.00	6.05	4.46	3.78	3.44
Poland	Middle childhood	5.89	10.43	13.01	9.08	22.74	13.33	9.70	4.80	4.89	6.13
Poland	Late childhood	5.28	7.24	10.10	5.80	20.72	21.34	11.50	5.63	5.40	6.98
Poland	Adolescence	4.32	8.23	7.91	11.86	25.26	7.78	7.98	6.79	13.81	6.05
Portugal	Late childhood	6.67	9.93	10.73	10.09	15.82	14.53	8.17	10.29	7.89	5.89
Switzerland	Middle childhood	9.33	9.93	9.94	9.15	15.32	15.64	6.93	8.73	8.20	6.83
Switzerland	Late childhood	6.39	5.08	12.13	5.79	29.83	21.99	5.14	5.87	3.76	4.02

Supplemental Material (SM) 5. Latent growth curve models: Fit measures

Country	Age group	Fit measure	Self-direction	Stimulation	Hedonism	Achievement	Power	Security	Conformity	Tradition	Benevolence	Universalism
Israel Arab citizens	Adolescence	RMSEA	0	0	0	0.05	0	0	0	0.07	0.09	0.08
		CFI	1	1	1	0.98	1	1	1	0.96	0.86	0.95
		SRMR	0	0	0.01	0.02	0.01	0.01	0.02	0.03	0.04	0.03
Israel Jewish majority	Middle childhood	RMSEA	0	0	0.07	0	0	0.07	0	0.02	0.1	0
		CFI	1	1	0.94	1	1	0.94	1	0.99	0.89	1
		SRMR	0.05	0.03	0.06	0.04	0.04	0.07	0.03	0.05	0.06	0.05
Israel Jewish majority	Adolescence	RMSEA	0	0	0	0	0	0	0	0.02	0.02	0.03
		CFI	1	1	1	1	1	1	1	1	1	1
		SRMR	0	0.01	0	0.01	0.01	0.01	0.01	0.01	0.01	0.02
Italy	Late childhood	RMSEA	0.06	0.03	0.02	0.07	0.09	0.05	0.03	0.06	0.02	0.08
		CFI	0.96	0.99	0.99	0.96	0.96	0.99	0.99	0.97	1	0.96
		SRMR	0.05	0.05	0.04	0.07	0.04	0.05	0.04	0.05	0.04	0.05
Poland	Middle childhood	RMSEA	0.06	0.06	0.07	0.03	0.07	0.02	0.06	0.04	0.03	0.04
		CFI	0.79	0.91	0.66	0.97	0.91	0.98	0.86	0.95	0.96	0.96
		SRMR	0.08	0.07	0.09	0.06	0.1	0.07	0.08	0.07	0.07	0.06
Poland	Late childhood	RMSEA	0.02	0.06	0.06	0.04	0.06	0.03	0.05	0.06	0.06	0.08
		CFI	0.99	0.96	0.92	0.98	0.96	0.99	0.96	0.97	0.94	0.93

		SRMR	0.04	0.07	0.07	0.06	0.06	0.06	0.06	0.06	0.08	0.07
		RMSEA	0.04	0.03	0.03	0.02	0.04	0.04	0.03	0.02	0.03	0.04
Poland	Adolescence	CFI	0.98	0.99	0.99	0.99	0.99	0.98	0.98	0.99	0.99	0.99
		SRMR	0.04	0.03	0.03	0.03	0.03	0.04	0.04	0.02	0.03	0.04
		RMSEA	0	0.04	0	0.07	0.02	0.07	0	0	0	0
Switzerland	Middle childhood	CFI	1	1	1	0.98	1	0.98	1	1	1	1
		SRMR	0.01	0.01	0.01	0.02	0.01	0.02	0	0	0	0
		RMSEA	0.05	0.13	0.11	0.06	0	0.11	0.14	0	0.04	0
Switzerland	Late childhood	CFI	0.99	0.97	0.96	0.99	1	0.96	0.93	1	0.99	1
		SRMR	0.03	0.03	0.04	0.03	0	0.04	0.05	0.01	0.02	0.02
